# The Limits to Sustainability Science: Ecological Constraints or Endless Innovation?

**DOI:** 10.1371/journal.pbio.1001343

**Published:** 2012-06-19

**Authors:** Georgina M. Mace

**Affiliations:** Department of Life Sciences, Imperial College London, London, United Kingdom

## Abstract

Ecological principles must govern sustainability, yet sustainability science is largely concerned with social-environmental interactions and barely considers physical limits on resource use. Whether it is possible to overcome such limits can be contested, but the issues raised by a macroecological perspective should be a fundamental part of the United Nations Conference on Sustainable Development (Rio+20).

The United Nations Conference on Sustainable Development (Rio+20) takes place in Rio de Janeiro on 20–22 June 2012. Twenty years after the 1992 Earth Summit that led to the establishment of two major environmental conventions (the United Nations Framework Convention on Climate Change and the Convention on Biological Diversity), Rio+20 presents an opportunity for the leaders of the world's governments to re-examine their commitments to sustainable development. An Essay by Burger et al. [1] in this issue and a Perspective contributed in response by Matthews and Boltz [2] raise concerns that certainly should be considered in Rio. But it's almost certain they won't be.

Burger et al. [1] present the case that the macroecology of sustainability is woefully under-represented in sustainability science. Ecological principles must govern sustainability, yet sustainability science is largely concerned with social–environmental interactions and barely considers physical limits on resource use. Escalating rates of resource use per capita, along with an increasing human population and environmental change, must, they argue, lead to limits in the availability of energy and materials on which the world's continuing economic development depends. Matthews and Boltz do not contest the evidence presented by Burger et al., but they are more optimistic that human ingenuity and adaptability will both buy time and provide solutions that will allow human societies to overcome resource limitation and continue to grow. Specifically, they contend that, despite the geometric increase in both population and resource use, a societal transformation is under way based around flexible, green economies that are in turn based in dynamic, variable ecosystems. They further argue that environmental pessimism will have less traction in policy-making than providing positive and creative approaches to these awkward problems.

This discussion is not new. Two issues have continued to be debated over the 20 years since the first Rio Earth Summit. One concerns the concept of sustainability and what it means in practice. A common query that has no easy answer asks about the sustainability of what, for whom, where, and over what time scales? Endless rhetoric about sustainable consumption and sustainable development hardly ever confronts the reality that, in most cases, what is sustainable for one sector of human society at one time and place rarely has no impact on other resources, or on environmental processes separated in time and space. The second theme, now discussed for over 40 years, is about the limits to growth. Any sensible person will agree that growth cannot continue indefinitely in a finite world. Yet over recent decades, the evidence indicates continuing growth, often at close to exponential rates in both population and consumption. How is this possible? Are we borrowing from the future, are we using resources that are far from their limits, or are we adapting creatively through innovation and technologically driven efficiency and replacement? Or, are we actually failing to act responsibly given evidence that certain limits are dangerously close, or even are already transgressed? [3]

Burger et al. present the argument for macroecological limits based on three inter-related themes and the evidence behind them. First, they describe how the flow of resources from the environment to support human societies must conform to physical laws concerning matter and energy. Therefore, at any spatial scale, flows of energy and nutrients for production and growth must come from somewhere, and a positive balance in one context will be felt as a negative balance somewhere else. Since smaller human systems (e.g., in towns and villages) are embedded in larger environmental systems, these flows and fluxes eventually add up to the global scale, where the finite nature of the biosphere and earth system must ultimately set limits. In fact, for the systems and resources that Burger et al. examine, there is evidence that we may already be reaching these limits. In the case of what is clearly a well-managed salmon fishery, resource flows have significant impacts on other components of the ecosystems (e.g., reduced resources for predators or decomposers). In what is an apparently sustainable urban system, the environmental costs to the surrounding landscape or on ecosystems elsewhere are shown to be substantial. In showing how per capita consumption of many materials and resources is now declining, Burger et al. suggest that their data may be the first evidence that we are approaching limits for some resources such as phosphorous, arable land, and freshwater. Some of this decline may be due to efficiencies, redundancy, and technological replacement of resources by innovative human societies, as Matthews and Boltz describe, but they agree that, ultimately, global constraints exist.

There is no doubt that these are critical issues for the environmental sciences to address. The research questions are difficult to pin down because they are embedded in a complex nexus of issues where ecological and evolutionary sciences, natural resource management, poverty alleviation, equitable and sustainable growth, individual rights and responsibilities, and the governance of the environment all converge. The academic community is increasingly engaged in defining the agenda for new science that will be needed. For example, following the recent Planet under Pressure meeting held in London, scientists sent a declaration to the Rio+20 conference [4] stressing that society is taking substantial risks by delaying urgent and large-scale action for environmental sustainability, and calling for a new approach to research that is more integrative, international, and solutions-oriented. In a similar vein, the 2012 Royal Society report on People and the Planet [5] concludes that rapid and widespread changes in the human population, coupled with unprecedented levels of consumption, present profound challenges to human health and wellbeing with important implications for future life on our finite planet.

The difference between ecological pessimism in Burger et al. and technological optimism in Matthews and Boltz is only one of the many ways that the problem can be viewed. Often the focus needs to be on extremes, or on non-linearities and irreversibilities in environmental systems that do not sit easily in standard economic analysis [6]. For example, species and ecosystems may be affected more by increases in the frequency of climate extremes than by shifts in mean values of temperature and precipitation. At a societal level, average rates of growth and development, both within and between countries, hide enormous disparity between the very rich and the very poor. The number or proportion of people living in extreme poverty is the key concern for development, not the average level of development, which is often the statistic of choice for scientific assessment and national reporting. More affluent societies tend to be more unequal, and inequality is itself an indicator of low wellbeing [4]. Similarly, while changes to some environmental resources are reversible with good restorative management, for many more, changes produce outcomes that are hard to predict (e.g., species responses to climate change), incur long time lags to recovery (e.g., recovery of fisheries following over-harvesting), or allow recovery but to an altered state (e.g., freshwater lakes following recovery from eutrophication) [7]. Non-linearities are a particular problem for resource management, where flows of resources that contribute to production, and constitute one element of national accounting via gross domestic product, take no account of the condition of stocks or resources. However, when resources are close to being depleted or exhausted, prices rise, pressures may increase, and complete collapse of the resource becomes more likely [8]. In some other cases, such as the extinction of species or the loss of biomes and biodiversity, the loss is irreversible.

Sustainability science therefore needs much stronger connections with environmental sciences, including macroecology. Green economies, a major focus for Rio+20, similarly need to be embedded in ecological principles and not simply be focused on economic growth based on new, greener production systems. Hopefully, in another 20 years, we can celebrate successful outcomes from the emergence of this integrated science for the environment and people.
